# Nasal Delivery of Cinnarizine Thermo- and Ion-Sensitive In Situ Hydrogels for Treatment of Microwave-Induced Brain Injury

**DOI:** 10.3390/gels8020108

**Published:** 2022-02-10

**Authors:** Yuanyuan Zhang, Qian Li, Jinglu Hu, Chunqing Wang, Delian Wan, Qi Li, Qingwei Jiang, Lina Du, Yiguang Jin

**Affiliations:** 1 School of Pharmacy, Shandong University of Traditional Chinese Medicine, Jinan 250355, China; 17853464723@163.com (Y.Z.); 17862969577@163.com (Q.L.); wcq19960911@163.com (C.W.); delian169@163.com (D.W.); qili1029@163.com (Q.L.); 2 Department of Pharmaceutical Sciences, Beijing Institute of Radiation Medicine, Beijing 100850, China; hujinglu187@163.com (J.H.); jinyg@bmi.ac.cn (Y.J.); 3 School of Pharmacy, Henan University, Kaifeng 475004, China; 4 Key Laboratory of Natural Medicine of the Changbai Mountain, Ministry of Education, College of Pharmacy, Yanbian University, Yanji 133002, China

**Keywords:** microwave-induced brain injury (MIBI), cinnarizine, sulfobutyl-β-cyclodextrin, inclusion complex, thermo- and ion-sensitive hydrogels, brain targeting, intranasal administration

## Abstract

(1) Background: When the body is exposed to microwave radiation, the brain is more susceptible to damage than other organs. However, few effective drugs are available for the treatment of microwave-induced brain injury (MIBI) because most drugs are difficult to cross the blood–brain barrier (BBB) to reach the brain. (2) Methods: Nasal cinnarizine inclusion complexes with thermo-and ion-sensitive hydrogels (cinnarizine ISGs) were prepared to treat MIBI and the characteristics of the inclusion complexes and their thermo-and ion-sensitive hydrogels were evaluated. (3) Results: Due to high viscosity, cinnarizine ISGs can achieve long-term retention in the nasal cavity to achieve a sustained release effect. Compared with the model, the intranasal thermo-and ion-sensitive cinnarizine ISGs significantly improved the microwave-induced spatial memory and spontaneous exploration behavior with Morris water maze and open field tests. Cinnarizine ISGs inhibited the expression of calcineurin and calpain 1 in the brain, which may be related to the inhibition of calcium overload by cinnarizine. (4) Conclusion: Intranasal thermo- and ion-sensitive cinnarizine ISGs are a promising brain-targeted pharmaceutical preparation against MIBI.

## 1. Introduction

Microwave radiation, which refers to electromagnetic waves with a frequency of 300 MHz to 300 GHz, has various effects on many organs of the body, and the brain is generally considered to be the most susceptible organ for microwave radiation [1]. Operators who have been chronically exposed to microwave radiation will experience memory loss, headache, dreams, insomnia, and other symptoms [2,3,4]. The mechanisms of microwave radiation-induced brain injury remain unclear, possibly involving neuronal degeneration, apoptosis and necrosis, mitochondrial swelling and cavitation, reduction in Nissl bodies [5,6], destruction of the blood–brain barrier [7,8], synaptic structure and function of plastic damage [9], and calcium overload [10]. The current methods for protection against MIBI are mostly based on physical protection, such as protective clothing and protective helmets, with fewer therapeutic drugs. Kangfuling [11], Anduolin [12], and other traditional Chinese medicine compound oral preparations have systemic effects without brain specificity.

Normally, the extracellular free Ca^2+^ concentration is much higher than the intracellular concentration. After microwave radiation, the intracellular calcium ion content increases significantly, causing energy metabolism disorders and normal physiological processes [13,14]. Therefore, calcium channel blockers may become effective drugs for MIBI, with these including selective Ca^2+^ channel blockers, such as nifedipine, nimodipine, and diltiazem, and non-selective Ca^2+^ channel blockers, such as cinnarizine and flunarizine. These blockers are characterized by the ability to inhibit the influx of extracellular calcium ions by blocking the calcium ion channels on cell membranes so that the vascular smooth muscles can be relaxed, the peripheral vascular resistance reduced, and the pharmacological effect of lowering blood pressure achieved. These drugs are not only used for the treatment of high blood pressure clinically but they are also used for the treatment of coronary heart disease and angina pectoris [15]. There is the possibility that Ca^2+^ channel blockers can be used as promising therapeutic drugs against MIBI.

Cinnarizine is a well-known Ca^2+^ channel blocker; our previous research showed that cinnarizine had superiority in improving the spatial memory capacity of rats and reducing neuronal damage in the hippocampus of rats. However, it is a weakly alkaline drug that is only soluble in solutions with a pH value of 1 or less, and its solubility drops sharply in solutions with a pH value of 3 or higher, which leads to significant individual differences and poor bioavailability during clinical oral administration [16]. Cyclodextrin is characterized by the ability to increase the solubility of poorly soluble drugs in water and to improve the bioavailability, and sulfobutyl-β-cyclodextrin (SBE-β-CD) has special affinity and inclusion characteristics for nitrogen-containing drugs.

Brain-targeted drug delivery is an important means by which to prevent and treat brain diseases through the BBB. Current intravenous injection methods involve the design of various nanoparticles that are complex and unsafe. Moreover, the drug dosage is restricted. All this poses an obstacle to simple or long-term use and to safe intracerebral drug delivery in case of MIRI. In situ hydrogels (ISGs) are fluid liquids without stimuli but they become semi-solid gels under the influence of some factors (such as temperature, pH, and ions) after administration. After the stimulus is removed, they can reversibly return to the initial state [17,18,19]. In situ gel has many advantages, such as simple preparation, easy administration, good patient compliance, long local residence time, and low irritation. The nasal cavity has a large superficial area and rich capillaries where the temperature is maintained at about 35.5 °C and the nasal fluid is rich in cations (Na^+^, K^+^, Ca^2+^) [20], making it an ideal area for the administration of thermo- and ion-sensitive hydrogels. The unique trigeminal nerve and olfactory nerve pathways in the nasal cavity provide a new strategy for brain-targeted delivery.

Here, SBE-β-CD was used to prepare the cinnarizine inclusion complexes to improve its solubility and bioavailability before it was loaded into thermo- and ion-sensitive ISGs consisting of poloxamer 407 (P407) and deacetylated gellan gum (DGG) [21]. As the temperature rose, P407 molecules aggregated to form micelles, and more gelation occurred. Moreover, DGG combined potassium and calcium ions in the nasal fluid to form thermo- and ion-sensitive ISGs that not only improved their bioavailability but could be administered through the nasal cavity to achieve brain targeting, whose MIBI pharmacodynamics were elucidated in an MIRI animal model.

## 2. Results and Discussion

### 2.1. Formation of Cinnarizine Inclusion Complexes

**Figure 1:**
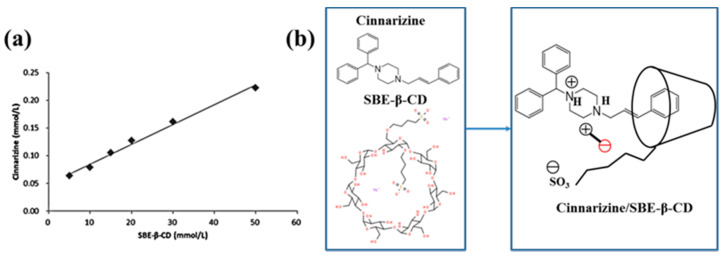
(**a**) Phase dissolution curves of cinnarizine and SBE-β-CD. (**b**) Inclusion mechanism of cinnarizine and SBE-β-CD.

A phase solubility curve is often used to judge the inclusion ratios of drugs and cyclodextrins. The concentration of cinnarizine was positively correlated with the increase in SBE-β-CD concentration (Figure 1a). This was in line with the solubility diagram of the A_L_ type phase, showing the formation of inclusion complexes at a 1:1 molar ratio [22,23] (Figure 1b).

We compared two methods of preparing cinnarizine inclusion complexes: grinding and freeze-drying. The water solubility of free cinnarizine was almost 0, while the water solubility of the cinnarizine inclusion complexes prepared with the grinding and freeze-drying methods was 4.10% (*w*/*v*) and 4.17% (*w*/*v*), respectively. Although their water solubility was similar, the preparation of the inclusion complexes by the freeze-drying method was relatively simple and not subject to human interference at an inclusion rate of 72.44%.

**Figure 2:**
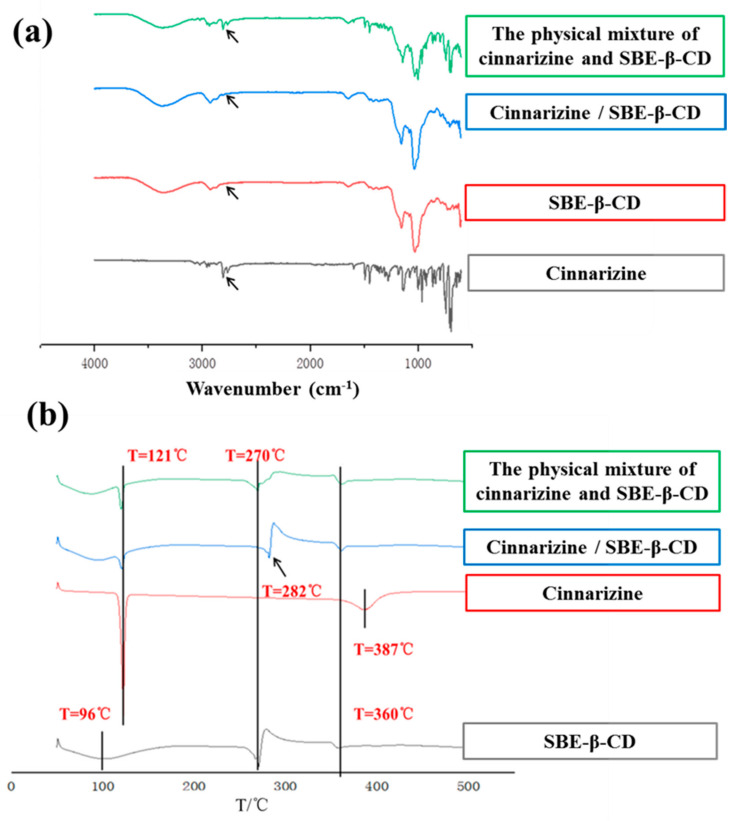
The infrared spectra (**a**) and DSC curves (**b**) of cinnarizine, SBE-β-CD, the cinnarizine SBE-β-CD inclusion complexes, and their physical mixture.

Infrared (IR) spectroscopy can be used to determine whether the inclusion complexes are formed. The skeletal vibration of the benzene ring of cinnarizine was at 1596 and 1680 cm^−1^. The stretching vibration peak of the hydroxyl group of SBE-β-CD was at 3203 cm^−1^. In contrast, the IR spectrogram of the cinnarizine inclusion complexes was much similar to that of SBE-β-CD, with the hydroxyl group peak of 3219 cm^−1^. Meanwhile, the characteristic peak of the benzene ring of cinnarizine disappeared. It showed that cinnarizine had been completely encapsulated in the cavity of SBE-β-CD. The IR spectroscopy of the physical mixture of cinnarizine and SBE-β-CD included both the characteristic peaks of cinnarizine and SBE-β-CD, which suggested that cinnarizine could not be trapped in the cavity of SBE-β-CD by straightforward physical blending (Figure 2a).

The peaks in the Differential Scanning Calorimeter (DSC) were also used to determine whether the inclusion complexes were formed. In the DSC curve, cinnarizine had a peak at 121 °C, compared with 270 °C for SBE-β-CD, which may be related to its thermal decomposition needing to absorb heat. There were small peaks of the physical mixture of cinnarizine and SBE-β-CD at 96 and 270 °C; however, there were small peaks of cinnarizine inclusion complexes at 96 and 282 °C. As the higher stability of the inclusion complex requires more heat to be absorbed for decomposition, the peak at around 270 °C was shifted to the right compared with the physical mixture (Figure 2b).

### 2.2. Characteristics of Cinnarizine ISGs

The P407 used in this research is a widely used thermo-sensitive polymer material, commonly used to prepare ISGs for the delivery of various drugs via ocular [24], nasal [25], vaginal [26], rectal [27], subcutaneous [28], topical [29], and intratumoral routes [30]. P407 has been approved by the FDA for ophthalmic or topical preparations due to its safety. Moreover, ISGs are used for stem cell therapy and regenerative medicine. The DGG solution can gel at a lower cation concentration because the cations can shield the electrostatic repulsion between the free carboxyl groups of DGG and improve the intermolecular cross bonding of DGG [31].

**Figure 3:**
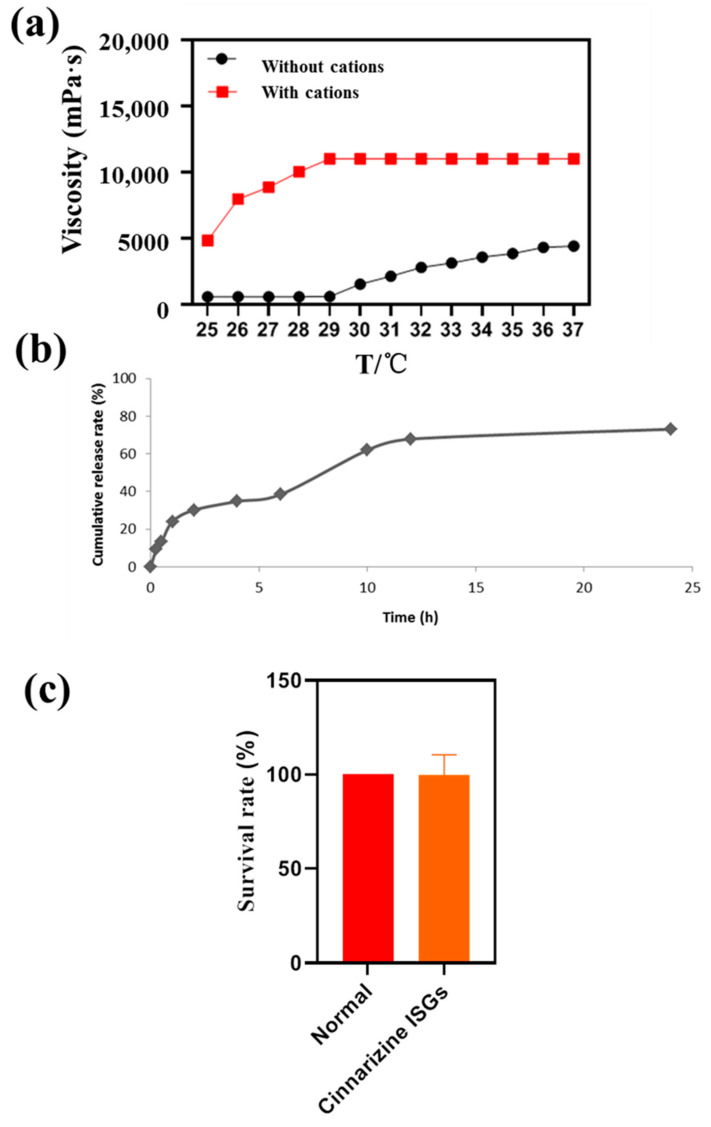
(**a**) Viscosity profiles of ISGs with temperature. (**b**) In vitro drug release profile of cinnarizine ISGs. (**c**) Survival rate of PC12 cells with cinnarizine ISGs.

The viscosity of medicine is very important for nasal drug delivery systems and the appropriate viscosity of nasal preparations should be about 5.0 Pa·s [32]. The viscosity of ISGs containing 0.3% (*w*/*v*) DGG and 17% (*w*/*v*) P407 was 5.05 Pa·s at 37 °C (Figure 3a), suitable for nasal drug delivery. The addition of Ca^2+^, K^+^ and Na^+^ improved the viscosity of the thermo- and ion-sensitive ISGs to 10.05 Pa·s.

### 2.3. Sustained Release and Cytotoxicity of Cinnarizine ISGs

It is common knowledge that ISGs with hydrophilic three-dimensional networks formed by chemical or physical cross-linking can be regarded as the perfect substrate for controlling drug release. In this research, the cumulative release rate of cinnarizine from ISG within 12 h was 75%, which was a slow-release process (Figure 3b). The mathematical simulation of the release curves suggested that the correlation coefficients of zero-order, first-order, and Higuchi equations were 0.770, 0.884, and 0.917. The release curve of cinnarizine from ISGs conformed to the Higuchi equation [33], indicating that the release mechanism of cinnarizine from ISGs was controlled by diffusion and matrix corrosion. This release mechanism of ISGs is preferred for facilitating long-term topical retention and release in the nasal cavity. Moreover, ISGs with SBE-β-CD might promote the cumulative permeation amount [34].

The survival rate of PC12 cells with cinnarizine ISGs was determined to evaluate the irritation (Figure 3c). This showed that cinnarizine ISGs were non-toxic to PC12 cells in 24 h.

### 2.4. High Brain Targeting of Intranasal Cinnarizine ISGs

**Figure 4:**
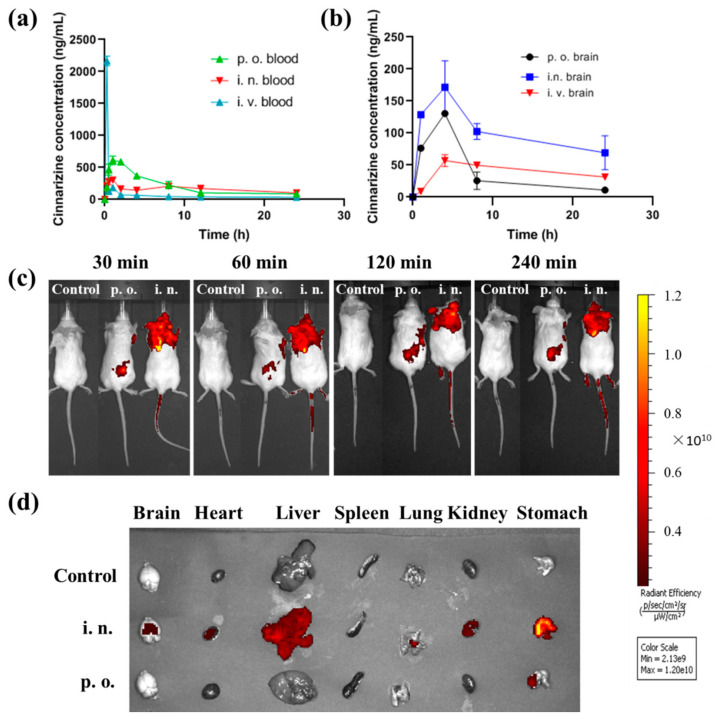
In vivo profiles of cinnarizine ISGs. (**a**) Cinnarizine formulations’ concentration in the blood by p.o., i.n., and i.v. administration (*n* = 5); (**b**) its concentration in the brain after p.o., i.v., and i.n. administration (*n* = 3). (**c**) In vivo fluorescence imaging of mice with RB via oral or nasal route over time. (**d**) Fluorescence imaging of the major organs at 240 min.

The in vivo process of intranasal cinnarizine ISGs and oral cinnarizine was compared. Intranasal cinnarizine ISGs had a T_max_ of 0.9 ± 0.2 h and a C_max_ of 304.2 ± 58.5 ng·mL^−1^, while p.o. had a T_max_ of 1.5 ± 0.44 h and a C_max_ of 611.7 ± 307.5 ng·mL^−1^ (Table 1, Figure 4a). Although the bioavailability of intranasal cinnarizine ISGs was less than the oral route, they had higher drug content in the brain (Figure 4b).

In this study, the brain targeting index (BTI, Equation (1)) was used to evaluate the brain targeting of the formulations. When the BTI value of formulations is greater than 1, this indicates that the formulations have good brain targeting. The brain C_max_ of i.n. cinnarizine was much higher than that of i.v. cinnarizine (Figure 4b). The BTI of i.n. cinnarizine ISGs was 1.16 while the BTI of p.o. cinnarizine was only 0.35, indicating the high brain targeting ability of i.n. cinnarizine.

Brain targeting index (BTI) = (AUCbrain/AUCblood)i.n./p.o./(AUCbrain/AUCblood)i.v(1)

Brain targeting of the i.n. ISGs was confirmed by in vivo imaging. There was no fluorescence distribution of rhodamine B (RB) in the control group. The fluorescence of the p.o. group was concentrated in the stomach 30 min after administration, while the fluorescence of the i.n group was concentrated in the brain (Figure 4c) and then the fluorescence slowly spread to other parts of the body. After the organs were removed, we found that the fluorescence of the p.o. group was mainly concentrated in the stomach while the fluorescence of the i.n group was distributed in the brain and other organs (Figure 4d), which proved that intranasal administration could achieve brain targeting. It should be noted that 4 h after intranasal administration, the fluorescence of RB was also distributed in other organs, and especially in the stomach. This is because a small amount of RB gel can slowly enter the esophagus through the nasal cavity, reach the stomach, and spread to the whole body.

### 2.5. Significant Therapeutic Effects of Cinnarizine ISGs on MIBI Rats

#### 2.5.1. Morris Water Maze to Detect the Spatial Memory of MIBI Rats

**Figure 5:**
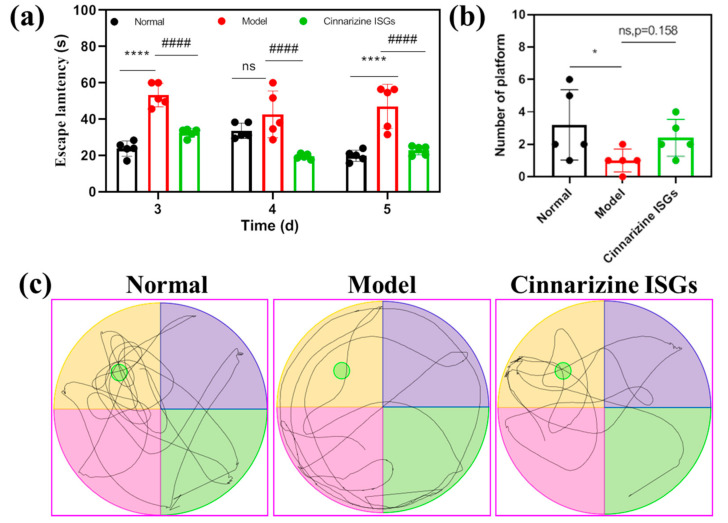
Average escape latency (**a**), number of platforms (**b**), and the trace map (**c**) of rats in the Morris water maze. (* *p* < 0.05, **** *p* < 0.001 vs. normal; #### *p* < 0.001 vs. model.)

On the 3rd and 5th days after microwave irradiation, the average escape latency (AEL) of rats in the model group was significantly higher than that of the healthy group (*p* < 0.05). This showed that the spatial memory of rats exposed to microwaves had been affected and these rats needed to take more time to find the escape platform. Compared with the rats in the model group, intranasal cinnarizine ISGs decreased the AEL of rats on the 3rd and 5th days after microwave irradiation, indicating that intranasal cinnarizine ISGs improved the spatial memory ability of rats exposed to microwave (*p* < 0.05, Figure 5a). In addition, intranasal cinnarizine ISGs improved the spontaneous exploratory capability of rats exposed to microwave compared to that of the model group, which can be proved by the trace of space exploration trials of rats and the increase in the times of crossing the escape platform (Figure 5b,c).

#### 2.5.2. Open Field Test to Evaluate the Spontaneous Behavior of MIBI Rats

**Figure 6:**
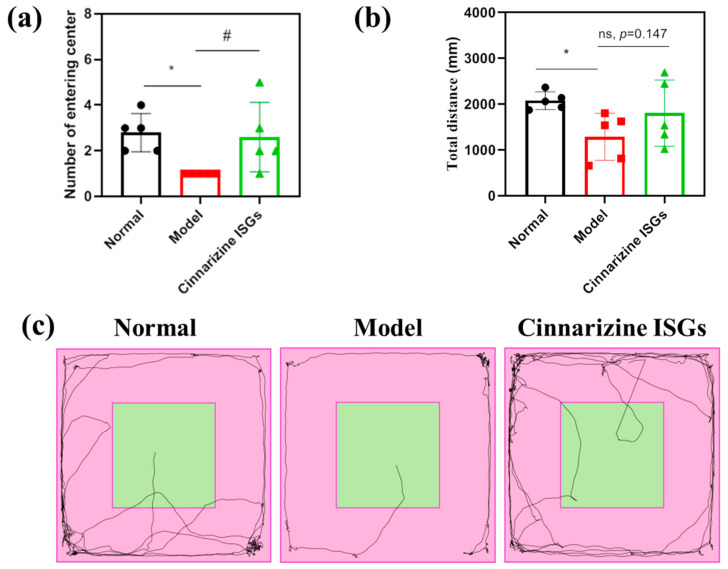
Number of times rats entered the central area (**a**), total distance (**b**), and the trace map (**c**) of rats in the open field. (* *p* < 0.05 vs. normal; # *p* < 0.05 vs. model.)

The total distance and the frequency of entering the central area were used as evaluating indicators to assess the spontaneous behavior of rats in the open field. The model rats displayed less spontaneous behavior than the healthy rats who were consistently moving in the surrounding area (Figure 6c).

The total distance (*p* < 0.05) and the number of times of entering the central area (*p* < 0.05) in the open field of MIBI rats were lower than those of the healthy group. The total distance and the number of times of entering the central area in the open field for the nasally administered cinnarizine ISGs groups (*p* = 0.147, *p* < 0.05) increased compared with the rats in the model group (Figure 6a,b), which indicated that cinnarizine ISGs could effectively improve the spontaneous behavior and curiosity of MIBI rats.

#### 2.5.3. Pathological Evaluation of MIBI Rats Treated with Different Strategies

The central nervous system (CNS) is sensitive to microwave irradiation, particularly the hippocampus [35,36]. After microwave irradiation, the cognitive function, learning, and memory of an organism associated with the hippocampus may be impaired [37]. Therefore, the hippocampus was the focus of our study of MIBI.

**Figure 7:**
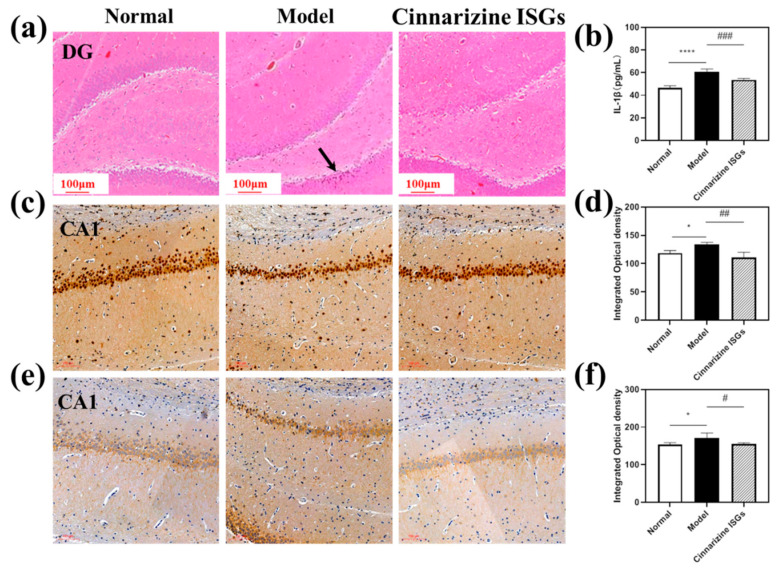
(**a**) H&E staining of the DG of the hippocampus in rat brains (the black arrows represent the pyknosis and deep staining parts of neurons); (**b**) expression of IL-1β in the rat hippocampus; (**c**) CaN expression of the CA1 of the hippocampus in the rat brain, and the integrated optical density (IOD) of the CA1 of the hippocampus in the rat brain (**d**); (**e**) calpain-1 expression of the CA1 of the hippocampus in the rat brain, and the IOD of the CA1 of the hippocampus in the rat brain (**f**). (* *p* < 0.05, **** *p* < 0.001 vs. normal; # *p* < 0.05, ## *p* < 0.01, ### *p* = 0.001 vs. model.)

The pyramidal neurons in the dentate gyrus (DG) region of the hippocampus of rats in the healthy group were neatly arranged and had regular karyotypes. In contrast, the cells in the DG region of the hippocampus of the rats in the model group were sparsely arranged, and the karyotypes were irregular in shape (triangular or fusiform) (Figure 7a). In addition, the nuclei of neurons in the model group contracted and were deeply stained, suggesting that microwave radiation could cause neuronal cell necrosis in the DG area of the hippocampus. Compared with the model group, the neurons in the cinnarizine ISGs group had no nuclear shrinkage and deep staining, which indicated that the pathological changes in various brain regions of the rats induced by microwave irradiation were significantly alleviated after nasal administration of cinnarizine ISGs.

The mechanism of microwave radiation damage to the hippocampus is very complex, including oxidative damage, blood–brain barrier damage, and calcium overload. Among them, the calcium signaling pathway plays a key role in memory formation [38].

The expressions of calcineurin (CaN) and calpain-1 can be used as a sign of calcium overload by stimulation. Compared to the healthy group, the yellow staining in the hippocampal CA1 region of the model group deepened, indicating that the expressions of CaN and calpain-1 increased significantly (Figure 7c,e). After seven days of administration, cinnarizine ISGs could reduce the expressions of CaN and calpain-1 in the hippocampal CA1 region.

Image J v1.8.0 (National Institutes of Health, Bethesda, MD, USA) software was used to analyze the integrated optical density (IOD) of each region and each group in immunohistochemistry. Compared to the healthy group, the expressions of CaN and calpain-1 in the CA1 area of the hippocampus in the model group were significantly unregulated (*p* < 0.05). However, cinnarizine ISGs (*p* < 0.05) could significantly reduce the expression of CaN and calpain-1 in the CA1 area of the hippocampus compared to the model group (Figure 6f and Figure 7d), which suggested that cinnarizine could treat microwave radiation brain injury by inhibiting calcium overload.

#### 2.5.4. Decreased Expression of IL-1β with Cinnarizine ISGs

After microwave irradiation, the content of IL-1β in the brain tissue of the model group was significantly higher than that of the healthy group (*p* < 0.05). Compared with the model group, cinnarizine ISGs (*p* < 0.05) could significantly reduce the content of IL-1β in rat brain tissue (Figure 7b), which suggested that inflammation may be one of the pathogenic mechanisms of MIBI.

### 2.6. Obvious Inhibitory Effects of Cinnarizine on Intracellular Ca^2+^ Content

**Figure 8:**
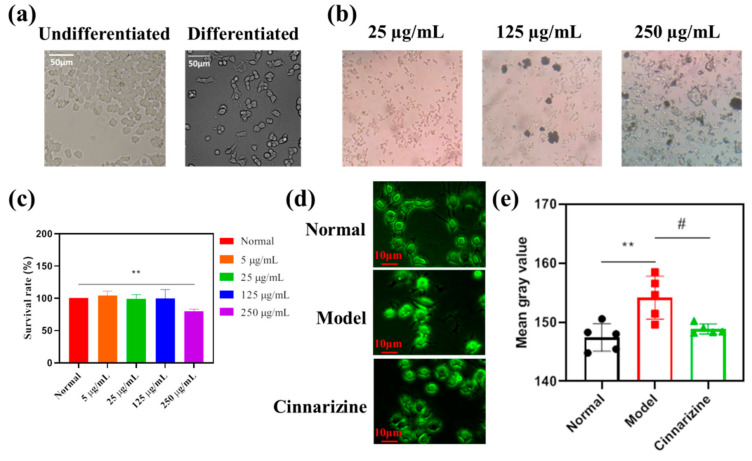
(**a**) Morphological changes in PC12 cells before and after differentiation (20×); (**b**) cell culture medium with cinnarizine (10×); (**c**) the cytotoxicity of PC12 cells with different concentrations of cinnarizine; (**d**) the pictures of PC12 cells with fluo-3; (**e**) the average fluorescence intensity of PC12 cells with fluo-3. (** *p* < 0.01 vs. normal; # *p* < 0.05 vs. model.)

As shown in Figure 8a, undifferentiated PC12 cells were observed to be round, and the synapses of NGF-inducting cells were extended gradually from both ends of the cell body. The pictures of the cell culture medium after adding cinnarizine are shown in Figure 8b and the cytotoxicity of PC12 cells with different concentrations of cinnarizine (0, 5, 25, 125, 250 25μg·mL^−^^1^) is shown in Figure 8c. After the addition of cinnarizine, the cell culture medium with 25 μg·mL^−^^1^ cinnarizine was clarified. For the CCK-8 assay (Figure 8c), exposure to 500 μg·mL^−^^1^ cinnarizine significantly decreased the cellular viability (*p* < 0.01), which was why 25 μg·mL^−^^1^ cinnarizine was added to evaluate the intracellular Ca^2+^ concentration after microwave radiation. After microwave radiation, the intracellular Ca^2+^ content increased significantly (*p* < 0.01), and the intracellular Ca^2+^ content decreased to the normal level after 25 μg·mL^−^^1^ cinnarizine was added (Figure 8d,e).

## 3. Conclusions

Inclusion technology is highly mature. The inclusion complexes formed by cinnarizine and SBE-β-CD significantly improved the solubility of the drug. ISGs are fluid liquids without outside stimuli but they become semi-solid gels under the influence of certain factors (such as temperature, pH, and ions). The thermo- and ion-sensitive cinnarizine ISGs prepared in this study had appropriate gelation temperature, quick gelation, and enough viscosity. These advantages proved that thermo- and ion-sensitive ISGs can achieve long-term retention in the nasal cavity to achieve a sustained release, which are ideal formulations for nasal delivery. Cinnarizine is a promising drug against MIBI, which may be related to the reduction in calcium overload. Moreover, the intranasal administration of ISGs can achieve brain targeting.

## 4. Materials and Methods

### 4.1. Materials 

The reagents used in this research are as follows: cinnarizine and SBE-β-CD (Beijing Innochem Science & Technology Co., Ltd., Beijing, China); P407 (Shanghai Yuanye Bio-Technology Co., Ltd., Shanghai, China); DGG (Tianwei Biochemical Engineering Co., Ltd., Zhuji, China); purified water and deionized water were prepared by the Heal Force Super NW Water System (Shanghai Canrex Analytic Instrument Co., Ltd., Shanghai, China).

### 4.2. HPLC Measurement

Cinnarizine was detected by a high-efficiency liquid chromatography (HPLC) system (Agilent 1260) with a C_18_ column (250 mm × 4.6 mm, 5 μm). The composition of the mobile phase was methanol with the mixture of 0.2% triethanolamine and 0.04% trimethylamine (pH = 6.6) at a ratio of 90:10 (*v*/*v*). Moreover, cinnarizine was detected at a detection wavelength of 254 nm, a column temperature of 30 °C, a flow rate of 1.0 mL/min, and a sample size of 10 μL.

### 4.3. Preparation of Cinnarizine Inclusion Complexes

Two methods were used for the preparation of cinnarizine inclusion complexes [39].

Grinding method: Cinnarizine was mixed with SBE-β-CD at mol ratios of 1:1, 1:2, and 1:3 in a mortar. A small amount of ethanol was added to the mortar to start one hour’s grinding in the same direction and obtain a paste-like mixture that was dried at a constant 40 °C and ground properly to uniformity.

Freeze-drying method: An appropriate amount of cinnarizine raw material was dissolved in 10 mL ethanol while SBE-β-CD was dissolved in 10 mL deionized water. Cinnarizine solution and SBE-β-CD solution were mixed at mol ratios of 1:1, 1:2, and 1:3 [40] and placed in a freeze dryer to be freeze-dried 42 h [39].

### 4.4. Characterization of Cinnarizine Inclusion Complexes

To study the inclusion rate of the inclusion complexes, the inclusion complexes prepared by the above methods were washed with ethanol three times (suction filtration) to remove free cinnarizine. After evaporation from light, they were dissolved in 10 mL of methanol and percolated with a 0.22 µm microporous membrane. The filtrate was diluted by an appropriate multiple and entered into an HPLC to measure the content of cinnarizine. A formula for calculating the inclusion rate is shown in Equation (2) [41]:

Inclusion rate (%) = Cinnarizine in inclusion complexes/Total Cinnarizine added × 100(2)

To study the solubility of the inclusion complex and cinnarizine in water, excessive cinnarizine and cinnarizine inclusion complex were added to deionized water and oscillated with 120 rpm at 37 °C for 7 d. Meanwhile, excessive cinnarizine was added to 0, 5, 10, 15, 20, 30, and 50 mmol/L SBE-β-CD solutions and oscillated with 120 rpm at 37 °C for 3 d. Then, the concentration of cinnarizine was detected by HPLC. A curve was drawn with the concentration of SBE-β-CD (X-axis) and the concentration of cinnarizine (Y-axis) to determine the type of inclusions.

An IR spectrogram of cinnarizine, SBE-β-CD, the cinnarizine/SBE-β-CD inclusion complexes, and their physical mixture were obtained 3 times with a Fourier Transform Infrared Spectrometer (Spectrum TWO, Shanghai, China) [42] at 4000~600 cm^−1^, using the Attenuated Total Reflection (ATR) approach. Meanwhile, their compatibilities had been studied by DSC at 50~500 °C (TA DSC Q2000, New Castle, England) [43]. During the test, the heating rate of the instrument was 10 °C/min, and N_2_ was used as the protective gas.

### 4.5. Preparation of Thermo- and Ion-Sensitive Cinnarizine ISGs

ISGs were prepared by the physical mixing of P407 and DGG [44]. DGG was added into deionized water (30 mL) and fully swollen at 80 °C. After DGG solution cooling at 4 °C, P407 and cinnarizine inclusion complexes were slowly added and mixed well. In order to obtain a pellucid solution, the resulting mixture was kept at 4 °C for 48 h and deionized water was added every 12 h to 50 mL. Here, we optimized the contents of DGG and P407 in ISGs through an orthogonal experiment. A series of hydrogels with DGG concentrations of 0.1, 0.2, and 0.3% (*w*/*v*) and P407 concentrations of 16, 17, 18, 20, and 22% (*w*/*v*) were prepared.

### 4.6. Optimization and Characteristics of Cinnarizine ISGs

The test tube inversion method was used to evaluate the gelation temperature of the ISGs. The ISGs were placed under a 20~40 °C water bath at a heating rate of 0.5 °C/min until ISGs’ gelation.

Artificial nasal fluids (ANF) contain 150 mM NaCl, 40 mM KCl, and 5 mM CaCl_2_, therefore the viscosity of ISGs containing 0.3% (*w*/*v*) DGG and 17% (*w*/*v*) P407 were determined at 25~37 °C with or without cations by the viscometer (DV-III ULTRA, Sydney, Australia).

### 4.7. In Vitro Release and Cytotoxicity of Cinnarizine ISGs

The cinnarizine ISGs (1 mL, 1 mg·mL^−^^1^) were put into a dialysis bag (cutting MW of 7000 Da) that was immersed in 20% ethanol solution of 50 mL and oscillated with 100 rpm at 37 °C. At different time points of 0.5, 1, 2, 4, 8, 12, and 24 h, 1 mL of release medium pipetted was used for HPLC analysis and 1 mL of 20% ethanol solution at 37 °C was immediately replenished [21,45]. There were three repeated experiments. The 20% ethanol solution was in line with the sink condition. The cumulative release rate (Q_n_) was calculated according to the following formula (3):

(3) $$Q_{n}=\frac{C_{n}\cdot V+V_{0}\sum_{i=1}^{n=1}C_{i}}{A}$$

where V (mL) was the volume of the release medium pipetted, C_n_ (μg·mL^−^^1^) was the concentration of Sample n, C_i_ (μg·mL^−^^1^) was the concentration of Sample i (i < n), V_0_ (mL) was the suction volume, and A (µg) was the drug content in the ISGs.

The drug release mechanism from ISGs was evaluated based on mathematical simulation models, inclusive of zero-order, first-order, and Higuchi equations. The equations were simulated by ELISA Calc (CSDN, Beijing, China). A high correlation coefficient (r) means a high correlation between simulated and real release mechanisms. The mathematical simulation model equations were listed as follows [46]:

(a) Zero-order model equation: $F_{t}=k_{\mathrm{ro}}t$
(b) First-order model equation: $\ln\left(1-F_{t}\right)=-k_{r1}t+\ln M$
(c) Higuchi model equation: $F_{t}=k_{H}t^{1/2}$

where F_t_ (%) was the cumulative release rate of the drug from ISGs for t (h).

The harvested PC12 cells were suspended in 0.1 mL hydrogel with 25 μg·mL^−^^1^ cinnarizine. The hydrogel was incubated at 37 °C with 5% CO_2_ in air for 24 h. In total, 100 μL of culture medium containing 10% CCK-8 was added to each well. Then, the pore plate was placed in the cell incubator and incubated for 2 h in a humidified atmosphere of 5% CO_2_, 37 °C. After 2 h, the absorbance was measured by the microplate reader at a wavelength of 450 nm.

### 4.8. Small Animal In Vivo Imaging

BALB/c male mice (20 ± 2 g) were given 20 μL of RB ISGs (4%) by nasal cavity (i.n.) or 0.2 mL of RB solution (0.4%) by gavage (p.o.), while the control group was not treated. After 30, 60, 120, and 240 min, the small animal in vivo imaging system (IVIS Spectrum) was used to observe the fluorescence distribution in the small animals under the excitation wavelength of 535 nm and the emission wavelength of 580 nm. Then, the mice were sacrificed by de-neck, and the heart, liver, spleen, lung, kidney, brain, and stomach were removed to observe the fluorescence distribution by IVIS Spectrum.

### 4.9. In Vivo Pharmacokinetic Studies and Cinnarizine Distribution in the Brain

Plasma and brain concentrations of cinnarizine were analyzed with an LC-MS/MS instrument that consisted of a C_18_ column (50 mm × 3.0 mm, 2.6 μm, Phenomenex, Technologies, Thermo Fisher Scientific, Waltham, MA, USA) at 30 °C. An LC system (LC-20AD, Shimadzu, Kyoto City, Kyoto Prefecture, Japan) coupled with a triple quadrupole MS/MS detector (API5000, AB Sciex, Framingham, MA, USA) was used. The mobile phase was composed of 0.1% formic acid aqueous solutions (A) and 0.1% formic acid acetonitrile solutions (B) at a flow rate of 0.6 mL/min and an injection volume of 2 μL. The gradient elution was as follows: 10% B from 0.0 to 0.3 min, 10–90% B from 0.3 to 1.4 min, 90% B from 1.4 to 1.8 min, 90–10% B from 1.8 to 1.9 min, and 10% B from 1.9 to 3.0 min.The analysis was operated in the multiple reaction monitoring (MRM) mode, and its MS parameters are shown in Table 2.

Cinnarizine was dissolved in acetonitrile to prepare cinnarizine solutions at concentrations of 10, 20, 50, 100, 200, 500, 1000, 2000, 5000, and 10,000 ng·mL^−1^. The above cinnarizine samples (5 μL) and the buspirone (Internal Standard, I.S.) in acetonitrile solution (50 ng·mL^−1^, 1 mL) were added into the blank plasma or the blank brain of 45 μL and homogenized with 0.9% saline (1:4, *w*/*v*) to obtain the final concentrations of 1, 2, 5, 10, 20, 50, 100, 200, 500, and 1000 ng·mL^−1^. Then, the mixture was vortexed for 1 min at room temperature and centrifuged with 14,000 rpm for 10 min at 4 °C. The plasma sample supernatant of 50 μL diluted with 50% acetonitrile aqueous solution of 200 μL and the brain homogenate sample supernatant of 20 μL diluted with 50% acetonitrile of 180 μL were used for analysis.

In the pharmacokinetics study, 45 rats were equally divided into 3 groups treated with the ISGs (i.n., 20 mg/kg), intravenous (i.v., 2 mg/kg), or oral (p.o., 20 mg/kg), respectively. At 0, 0.25, 0.5, 1, 2, 4, 8, 12, and 24 h, the rats’ blood was taken 300 μL from the tail vein and centrifuged at 4000 rpm for 15 min at 4 °C to obtain plasma. At 1, 4, 8, and 24 h, the rats were sacrificed and the whole brain tissues were taken out. The buspirone (I.S.) in acetonitrile solution (50 ng·mL^−1^, 0.6 mL) was added to the plasma and brain homogenate of 30 μL; then, the mixture was vortexed for 1 min at room temperature and centrifuged at 14,000 rpm for 10 min at 4 °C. The plasma supernatant of 50 μL diluted with 50% acetonitrile aqueous solution of 200 μL and the brain homogenate supernatant of 20 μL diluted with 50% acetonitrile of 180 μL were used for LC-MS/MS analysis.

### 4.10. Radiation Protection of Cinnarizine ISGs

#### 4.10.1. Animals and Groups

**Figure 9:**
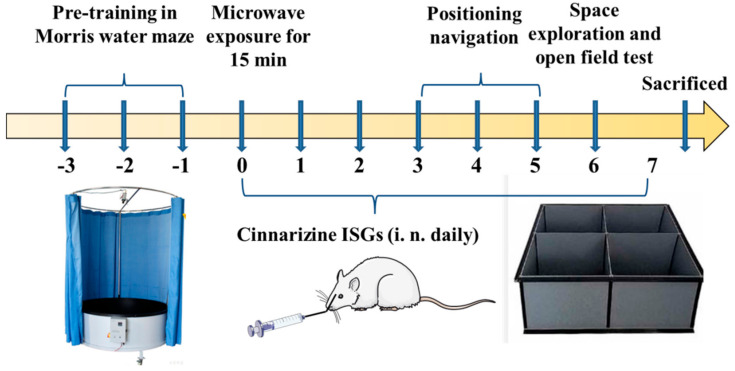
Scheme of pharmacodynamics study of cinnarizine ISGs.

Male Wistar rats weighing 180~220 g were purchased from Beijing SPF Science & Technology Co., Ltd., Beijing, China, which were of specific pathogen-free (SPF) grade and were maintained in an animal clean cabinet. All animal experimental procedures were conducted according to the guidelines of current laws and policies. The rats were divided into three groups with 8 rats in each group. Those radiated with 30 mW/cm^2^ for 15 min but left untreated served as the model group [34]. Rats that were topically administrated with 100 μL cinnarizine ISGs (60 mg·mL^−1^, 60 mg·kg^−1^·d^−1^, twice a day for seven consecutive days) by unilateral nasal cavity 1 h after radiation were referred to as the cinnarizine ISGs group. Those without radiation were classified as the control group (Figure 9).

#### 4.10.2. Morris Water Maze

A Morris water maze (MWM) (ZS-001, Beijing, China), with 150 cm diameter and an escape platform of 8 cm diameter, was used to test the learning and memory abilities of rats via location navigation, and space exploring tests [47]. The depth of the water in MWM was 1.5 cm higher than the escape platform and the water temperature was maintained at 28 ± 0.2 °C. The water area was segmented into four quadrants (Quadrants I–IV) on average, with the escape platform at the center of Quadrant III. Each rat was separately lowered into the water at the middle edge of Quadrants I-IV. The movement traces of the rats were automatically traced by Labmaze V3.0 tracking system (Beijing Zhongshidichuang Science and Technology Development Co., Ltd., Beijing, China). During three days of training, the rats who climbed on the platform within 60 s were allowed to stay on the escape platform for 20 s. If not, they were guided onto the escape platform and kept there for 20 s. The location navigation trials of rats were similar to the above training process but without guidance and carried out on the 3rd, 4th, and 5th days after i.n. administration of cinnarizine ISGs. The escape latency was defined as the time it took the rat to find the escape platform and kept there for more than 2 s after entering the water. If the rat failed to find the escape platform within 60 s, the escape latency was recorded as 60 s. The AEL of each rat was the average of the escape latency of the four quadrants.

The space exploration trials were carried out on the 6th day after i.n. administration of cinnarizine ISGs. The escape platform was removed, and the rats were placed into the water at the middle edge of the quadrant opposite that of the original escape platform. The number of times rats crossed the escape platform within 60 s was recorded.

#### 4.10.3. Open Field Test

The autonomous exploration behavior of the rats in a new environment was appraised by open field test (OFT) on the 6th day [48]. The open field (ZS-ZFT, Beijing Zhongshidichuang Science and Technology Development Co., Ltd., Beijing, China) was 100 cm long, 100 cm wide, and 50 cm high. All rats were put in the center of the open field on day 6 after administration of cinnarizine ISGs. The Labmaze V3.0 tracking system recorded the movement tracks of rats within 5 min.

#### 4.10.4. Pathological Observations and Expressions of IL-1β, CaN, the Calpain-1 Receptor in MIBI Rats

After behavioral evaluation, the rats were sacrificed by neck removal and the brains of rats were immersed in 4% paraformaldehyde. The brain was sectioned for H&E staining and CaN and calpain-1 immunohistochemistry. Hippocampal tissues of rats were peeled on ice. Hippocampal tissues/normal saline (1:9, *w*/*v*) were homogenized for 2 min and centrifuged at 4 °C at 1000× *g* for 10 min to obtain the supernatant by desktop freezing centrifuge (Fresco 21, Thermo Fisher Scientific Co., Ltd., Waltham, MA, USA). Then, the amount of IL-1β in the brain tissue was detected according to the instructions of the ELISA kit.

### 4.11. The Mechanism Evaluation—The Influence of Cinnarizine on Intracellular Ca^2+^ Content

#### 4.11.1. Cell Culture

PC12 cells were cultured in RPMI-1640 (1640) medium supplemented with 1% penicillin–streptomycin (100×), 10% horse serum, and 5% fetal bovine serum in a humidified atmosphere at 37 °C, 5% CO_2_ [49,50]. PC12 cells were inoculated on a Petri dish pre-coated with 0.1 mg·mL^−1^ poly-L-lysine (Sigma) at a density of 2.5 × 10^4^ cells/cm^2^ [51,52] and were differentiated into neuron-like cells with 1640 medium containing 1% horse serum and 50 ng·mL^−1^ nerve growth factor NGF-2.5S (Sigma) [53,54]. Half of the medium was renewed every other day. Four to five days later, the PC12 cells were observed under the microscope to judge the degree of differentiation.

#### 4.11.2. Microwave Radiation and the Influence of Cinnarizine on Intracellular Ca^2+^ Content

Differentiated PC12 cells were replaced with 1640 medium containing different concentrations of cinnarizine (0, 5, 25, 125, and 250 μg·mL^−1^), and the cell survival was detected by CCK-8 assay [55] to choose a suitable concentration.

The differentiated PC12 cells were exposed to continuous microwave radiation for 15 min with an average power density of 30 mW/cm^2^, and sham radiation control cells were treated under the same conditions without microwave exposure. The intracellular Ca^2+^ concentration was determined with a fluo-3/AM fluorescent probe, whose excitation wavelength was 488 nm. After incubation with cinnarizine (25 μg·mL^−1^), the PC12 cells were stained with fluo-3/AM (5 μM) for 40 min and observed under a fluorescence microscope.

### 4.12. Data Analysis

The data were expressed as means ± SD. Statistical differences between groups were analyzed with one-way ANOVA analysis of variance. All statistical analyses were performed by IBM^®^ SPSS^®^ Statistics software (Version 21). *p* < 0.05 indicated significant statistical differences between groups.

## Figures and Tables

**Table 1:** Brain targeting parameters of cinnarizine following intranasal and oral administration.

Administration Routes	Tissues	AUC_0–24_ (h*ng·mL^−^^1^)	C_max_ (ng·mL^−^^1^)	T_max_ (h)	AUC_brain_/ AUC_blood_
i.n.	Blood	3890	304.2	0.9	0.63
	Brain	2436	171.6	4	
p.o.	Blood	4945	611.7	1.5	0.19
	Brain	952.8	130.4	4	

**Table 2:** Multiple reaction monitoring (MRM) conditions.

Name	Q_1_ Mass (Da)	Q_3_ Mass (Da)	Time (Msec)	DP (Volts)	EP (Volts)	CE (Volts)	CXP (Volts)
Buspirone	386.4	122.2	100	180	11	43	16
Cinnarizine	369.3	167.2	100	20	11	20	4

## Data Availability

The data presented in this study are available on request from the corresponding author.
